# High erythropoietin levels are associated with low neurofilament light levels in simulated high altitude: a further hint for neuroprotection by erythropoietin

**DOI:** 10.3389/fneur.2025.1608763

**Published:** 2025-07-28

**Authors:** Klaus Berek, Anna Berek, Angelika Bauer, Dagmar Rudzki, Franziska Di Pauli, Gabriel Bsteh, Markus Ponleitner, Benedikt Treml, Axel Kleinsasser, Thomas Berger, Maria Wille, Martin Burtscher, Markus Reindl, Florian Deisenhammer, Harald Hegen

**Affiliations:** ^1^Department of Neurology, Medical University of Innsbruck, Innsbruck, Austria; ^2^Institute of Hygiene and Medical Microbiology, Medical University of Innsbruck, Innsbruck, Austria; ^3^Department of Neurology, Medical University of Vienna, Vienna, Austria; ^4^Comprehensive Center for Clinical Neurosciences and Mental Health, Medical University of Vienna, Vienna, Austria; ^5^Department of Anaesthesiology and Critical Care Medicine, Medical University of Innsbruck, Innsbruck, Austria; ^6^Department of Sport Science, University of Innsbruck, Innsbruck, Austria

**Keywords:** erythropoietin, high altitude, acute mountain sickness, neurofilament light, axonal damage

## Abstract

**Background:**

Erythropoietin (EPO) plays a crucial role in the early adaption to high altitude and is possibly involved in neuroprotection. Neurofilament light chain (NfL) is an established marker of neuroaxonal damage.

**Objective:**

To investigate whether EPO dynamics in simulated high altitude are linked to neuroaxonal damage as measured by NfL.

**Methods:**

Sixty-three healthy subjects were exposed to simulated altitude of 4,500 m for 12 h in a normobaric hypoxic chamber at the University of Innsbruck. Clinical data (heart rate, arterial oxygen saturation) were assessed before and 3 h after high altitude exposure; plasma samples were drawn before (measurement (M) 1) and after 12 h (M2). The levels of EPO and hypoxia-inducible factor (HIF)-1α were quantified using commercially available ELISA kits. NfL concentrations were measured using the Simoa SR-X Analyzer, and NfL Z scores calculated using age- and body mass index (BMI)-adjusted reference values.

**Results:**

EPO significantly increased after 12 h (M2: 10.12 [7.86–14.06] mU/mL vs. M1: 4.17 [2.99–5.67] mU/mL, *p* < 0.001), while HIF-1α did not significantly change (*p* = 0.409). Subjects with high EPO levels at M2 showed significantly lower NfL concentrations (5.85 [4.15–6.85] pg/mL vs. 6.73 [4.70–8.64] pg/mL, *p* = 0.030) as well as lower NfL Z scores (0.64 [−0.88–1.17] vs. 0.95 [0.25–1.48], *p* = 0.040) than those with low EPO levels. The extent of heart rate increase showed a positive correlation with EPO levels at M2 (r_s_ = 0.322, *p* = 0.011).

**Conclusion:**

Higher EPO concentrations were associated with lower NfL levels. This might further substantiate the hypothesis of a neuroprotective role of EPO.

## Introduction

Exposure to high altitude carries a risk due to various reasons including the decrease in oxygen partial pressure. Physiological ways to cope with hypoxia comprise increase of ventilation and heart rate, as well as production of red blood cells (RBC) (1–3). Nevertheless, this adaption is highly variable depending on the absolute height reached, the degree of acclimatization and the time to reach high altitude (4, 5). If adaption fails, high-altitude illness spanning a spectrum from acute mountain sickness (AMS) to high altitude pulmonary (HAPE) and/or cerebral edema (HACE) may occur (6, 7).

Erythropoietin (EPO) plays a key role in the adaption to high altitude hypoxia (3, 8) and acts as an anti-inflammatory mediator (9, 10). Its production is mediated by hypoxia-inducible factor (HIF)-1α (11). A neuroprotective effect of EPO has been proposed (10, 12, 13), even though data on this are contradictory (14, 15) and neuroprotection is difficult to measure.

Neurofilament light (NfL) is an established marker of neuroaxonal damage (16), which has proven its potential in several neurological diseases, including brain hypoxia (17). While there are studies on EPO levels in high altitude, data on a possible link between EPO and NfL levels as marker of neuroaxonal damage are lacking.

Therefore, we aimed to investigate whether EPO and NfL dynamics in simulated altitude are interlinked and may substantiate the proposed neuroprotective role of EPO.

## Methods

A detailed description of the study design has been previously published (18, 19). Briefly, a cohort of 63 healthy subjects was recruited at the University of Innsbruck. Subjects who had visited high altitude areas prior to the study (≥2,500 m for a daytrip within 14 days prior to study initiation, >24 h within the last month before study participation, or permanent residency in heights of ≥1,000 m) were not eligible. Also, subjects with reported history of neurological, cardiological, pulmonary or psychiatric diseases were excluded. For the present analysis, only remaining samples from this prior study were used (18, 19).

After a first check-up, all subjects stayed in a normobaric hypoxic chamber located at the Department of Sports Science, Leopold-Franzens University, Innsbruck, Austria, for 12 h simulating an approximate altitude of 4,500 m with an artificial oxygen level of 12.6%.

Demographic (age and sex) and basic clinical data [body mass index (BMI), heart rate in beats per minute (bmp), and arterial oxygen saturation (SaO2)] were assessed prior to entering the hypoxic chamber. Measurement of heart rate and SaO2 was repeated after 3 h in simulated high altitude. All heart rate and SaO2 measurements were performed standardized in sitting position. Symptoms and severity of AMS were assessed by the 2018 revised Lake Louise Acute Mountain Sickness Score (LLS). This scoring system ranges from 0 (no symptoms) to 12 (severe AMS symptoms) (20). AMS was defined as a maximal LLS score ≥ 4 (18, 20).

Blood (EDTA plasma) was sampled by a clinician before entering the hypoxic chamber (Measurement 1 [M1]) and 12 h later after exiting the chamber (M2).

### Measurement of analytes

Samples were stored at −20°C until measurement. EPO and HIF-1α were measured at the Medical University of Innsbruck under blinded conditions using commercially available ELISA kits from Thermo Fisher Scientific (Waltham, MA, United States), following the manufacturer’s instructions. For the EPO ELISA, samples were diluted to achieve a detectable range of 1.6–100.0 mU/mL. Each sample or diluted standard (50 μL) was incubated with 50 μL of biotin-conjugate for 1 h at room temperature with shaking. After washing, 100 μL of Streptavidin-HRP solution was added to each well and incubated at room temperature for 15 min on a shaker. Following another washing step, 100 μL of TMB substrate solution was added. The reaction was stopped after approximately 10 min of incubation at room temperature in the dark when the highest standard has reached an OD of 0.90–0.95. The absorbance was then read on a microplate reader at 450 nm with reference wavelength at 620 nm.

For the HIF-1α ELISA, 50 μL of undiluted samples or standards were added to each well and incubated for 2 h at room temperature with shaking. After washing, 50 μL of biotinylated detection antibody was added and incubated for 1 h at room temperature with shaking. After washing, 50 μL of streptavidin-HRP was added, which was incubated for 30 min at room temperature. After a final washing step, 100 μL of TMB substrate was added. After 30 min, the reaction was stopped with 100 μL of stop solution, and the absorbance was measured at 450 nm. In case of undetectable analyte levels, we used the lower detection limit (HIF-1α: 81.92 pg/mL; EPO: 1.60 mU/mL).

Plasma levels of Neurofilament light chain (pNfL) have already been determined previously (19). Briefly, pNfL was measured by single-molecule array (SIMOA) technique using the Simoa® NF-Light™ Advantage Kit (lot number: 503470) on the Simoa SR-X Analyzer (Quanterix, Lexington, MA, United States) (21). pNfL measurement was performed at the Medical University of Vienna under blinded conditions. The inter-assay coefficient of variation of pNfL was 12.4%.

To account for physiological variations of NfL levels, we calculated age- and BMI-adjusted NfL Z values according to Benkert et al. (22).

### Statistical analysis

Statistical analysis was performed using SPSS 26.0 (SPSS Inc., Chicago, IL, United States). Data were displayed as median and 25th; 75th percentile. Group comparisons were done by Mann–Whitney-U test, repeated measurements by Wilcoxon test. Spearman correlation coefficient (r_s_) was used for correlation analysis. According to median EPO levels/ median EPO increase subjects were stratified into low and high EPO groups. Two-sided *p*-values < 0.05 were considered statistically significant.

### Ethics

The ethics committee of the Medical University of Innsbruck approved this study (approval number 1130/2022). Written informed consent was obtained from all study participants.

## Results

A total of 63 previously recruited (19) individuals at a median age of 24 [22;28] years were included into the study. Twenty-seven (43%) were females, the median BMI was 22 [21;24] kg/m^2^. Detailed information on demographic, clinical and laboratory data of our cohort are given in Table 1. Of note, NfL levels were available from a previous analysis (19); HIF-1α and EPO concentrations were determined for the present study.

**Table 1:** Demographic, clinical, and laboratory data of participants.

Demographics	
Number of participants	63
Sex (female)	27 (43)
Age (years)	24 [22;28]
BMI (kg/m^2^)	22 [21;24]

	M1	M2	*p*-value
Laboratory characteristics			
Number of samples	63	63	
EPO (mU/mL)	4.17 [2.99;5.67]	10.12 [7.86;14.06]	**<0.001**
HIF-1alpha (pg/mL)	81.92 [81.92;159.51]	81.92 [81.92;194.83]	0.409
pNfL (pg/mL)	5.57 [4.39;8.01]	6.20 [4.54;7.64]	0.755
NfL Z score	0.41 [−0.28;1.37]	0.71 [−0.39;1.28]	0.631
Clinical characteristics			
Heart rate (bpm)	80 [73;86]^*^	84 [78;91]^*^	**0.001**
Heart rate Increase (bpm)		6 [−2;14]	n.a.
SaO2 (%)	97 [96;98]^*^	83 [80;87]^*^	**<0.001**
SaO2 Decrease (%)		14 [10;17]	n.a.
Highest LLS		3 [1;4]	n.a.

All values are depicted as median [25th; 75th percentile] and counts as *n* (%) as appropriate. *p*-values < 0.05 are marked bold. ^*^M2 of clinical data was performed after 3 h in simulated high altitude. LLS was assessed according to Roach et al. (20), NfL Z values were calculated according to Benkert et al. (22). BMI, Body mass index; bpm, beats per minute; HIF, Hypoxia induced factor; LLS, Lake Louise Acute Mountain Sickness Score; M, Measurement; n.a., not applicable; SaO2, arterial oxygen saturation.

### Clinical and biomarker changes associated with simulated high altitude exposure

Heart rate significantly increased 3 h after simulated high altitude exposure (M1: 80 [73;86] bpm, M2: 84 [78;91] bpm, *p* = 0.001), while SaO2 decreased (M1: 97 [96;98] %, M2: 83 [80;87] %, *p* < 0.001).

**Figure 1:**
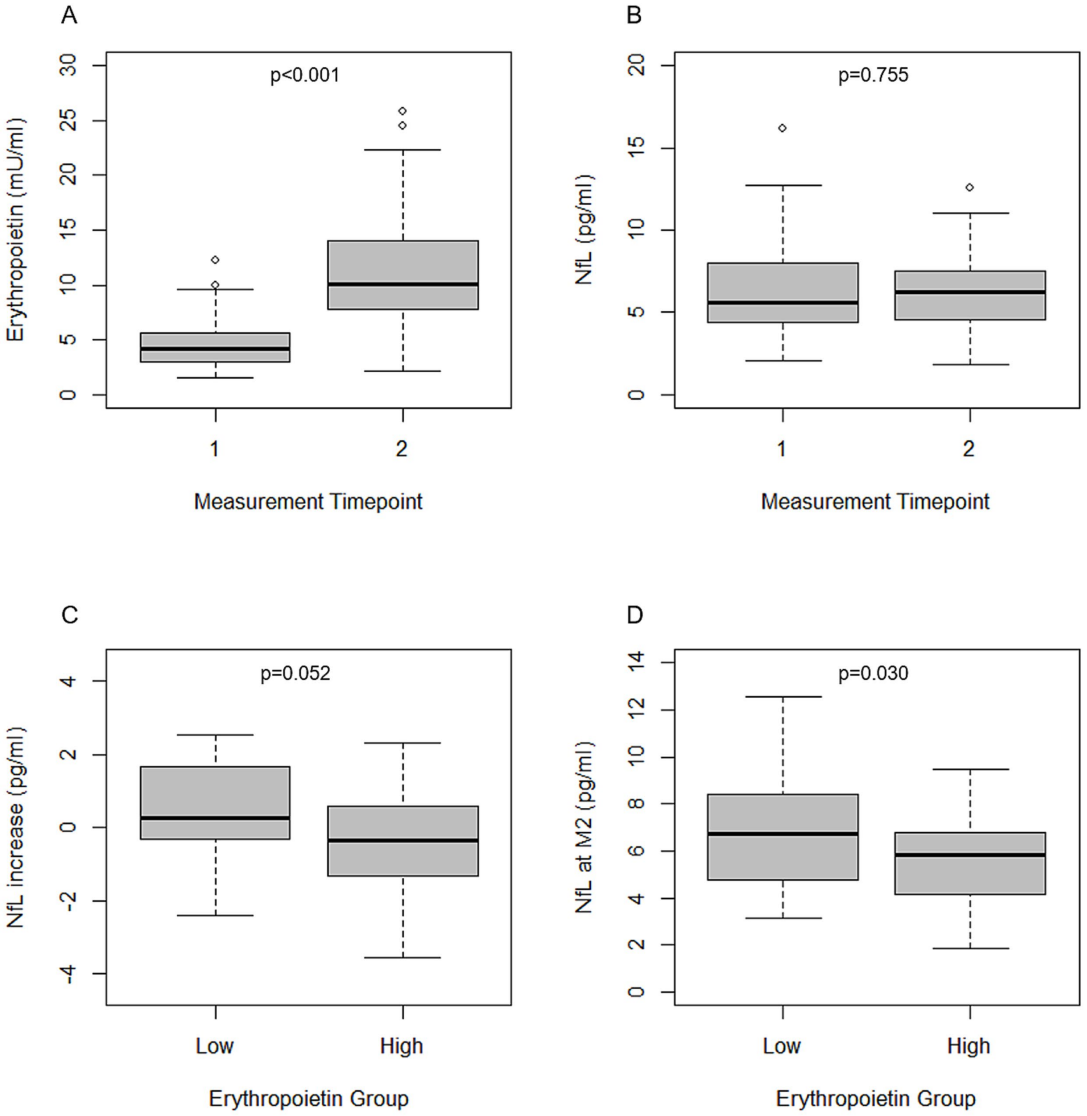
EPO and NfL concentrations before and after simulated high altitude exposure. **(A)** EPO levels before and 12 h after simulated high altitude. **(B)** NfL levels before and 12 h after simulated high altitude. **(C)** NfL increase between M1 and M2 in subjects within the low and high EPO group. Stratification into low and high EPO group was done according to the median increase of EPO concentrations between M1 and M2 (5.85 mU/mL). **(D)** NfL levels at M2 in subjects within the low and high EPO group. Stratification into low and high EPO group was done according to the median EPO concentration at M2 (10.12 mU/mL). EPO, Erythropoietin; NfL, Neurofilament Light.

Overall, EPO showed a significant increase 12 h after simulated high altitude exposure (M1: 4.17 [2.99;5.67] mU/mL, M2: 10.12 [7.86;14.06] mU/mL, *p* < 0.001, Figure 1A). Concentrations of HIF-1α (M1: 81.92 [81.92;159.51] pg/mL, M2: 81.92 [81.92;194.83] pg/mL, *p* = 0.409) as well as of absolute pNfL concentrations (M1: 5.57 [4.39;8.01] pg/mL, M2: 6.20 [4.54;7.64] pg/mL, *p* = 0.755, Figure 1B) and NfL Z scores (M1: 0.41 [−0.28;1.37], M2: 0.71 [−0.39;1.28], *p* = 0.631) did not show a statistically significant change.

### High EPO levels are associated with low NfL levels

However, the change of pNfL concentrations was different depending on the EPO levels. pNfL concentrations decreased between M1 and M2 in the high EPO group (−0.36 [−1.34;0.63] pg/mL), while there was an increase of pNfL in the low EPO group (0.26 [−0.41;1.68] pg/mL, *p* = 0.052, Figure 1C). NfL Z scores also decreased in the high EPO group (−0.20 [−0.83;0.26]) and increased in the low EPO group (0.21 [−0.07;0.53], *p* = 0.012). At M2 absolute pNfL concentrations and NfL Z scores were significantly lower in individuals with high EPO levels compared to those with low EPO (pNfL: 5.85 [4.15;6.85] pg/mL vs. 6.73 [4.70;8.64] pg/mL, *p* = 0.030, Figure 1D NfL Z score: 0.64 [−0.88;1.17] vs. 0.95 [0.25;1.48] *p* = 0.040).

### Physiological variables correlate with EPO increase

There was a positive correlation between the extent of the heart rate increase and EPO levels at M2 (r_s_ = 0.322, *p* = 0.011), while a negative correlation between SaO2 decrease and EPO levels (r_s_ = −0.276, *p* = 0.031). There was no correlation between the LLS score and EPO levels (r_s_ = −0.050, *p* = 0.702). EPO levels of individuals experiencing AMS at any point of the study did not differ from AMS-free individuals at M1 (4.72 [3.02;5.66] mU/mL vs. 3.83 [2.54;5.67] mU/mL, *p* = 0.289) and M2 (12.07 [7.86;15.85] mU/mL vs. 9.52 [7.84;12.78] mU/mL, *p* = 0.410).

## Discussion

In the present study, we provide data on EPO and NfL metrics before and after simulated high altitude exposure. There were two main findings: (i) EPO increased already 12 h after simulated high altitude exposure correlating with heart rate increase and SaO2 decrease, (ii) subjects with higher EPO increase showed lower NfL levels.

Worldwide, high altitude regions are visited for various reasons with the risk of developing high-altitude illness, of which AMS is the most common (7). The predominating cornerstone of AMS pathophysiology is hypoxia, caused by a low oxygen partial pressure and leading to expected oxygen saturation in healthy subjects of around 75–85% in 4,500 m (23, 24). EPO is crucial in the early adaption to high altitude (11) and during the adaption to hypoxia, respectively (3, 8). Of note, also a neuroprotective role of EPO has been proposed (12, 13), even though not undisputed. For instance, it has been reported that EPO application ameliorates the clinical and histological outcome in cuprizone-induced demyelination in mice (25), that EPO improves the survival and even regeneration of insect neurons (26), or that in a rat model of multiple sclerosis EPO increased the survival of retinal ganglion cells (27). From a clinical perspective, a recent multicenter study, investigating EPO as add-on therapy to mechanical thrombolysis during acute ischemic stroke, seems to be of special interest. Herein the first investigation showed a negative result, even if, an explorative subgroup analysis suggested that patients not receiving thrombolysis had a benefit from EPO (15). In a previous study body fluid biomarkers for brain damage after ischemic stroke including S100B, glial fibrillary acid protein (GFAP) and ubiquitin C-terminal hydrolase (UCH-L1) were significantly lower in the EPO treated patients than in placebo treated ones (14).

Here, we provide data of EPO and NfL levels measured in 63 healthy individuals before and after a 12 h-exposure to simulated high altitude in a normobaric hypoxic chamber. The first finding of our study, i.e., the significant increase of EPO already 12 h after simulated high altitude exposure is in line with existing literature. The temporal dynamics of biological markers in human organisms after high altitude exposure follow roughly the following schedule. First, within minutes to hours after the ascent, heart and ventilation rate as well as cerebral blood flow increase. Secondly, within hours to days EPO levels start to increase. It has been suggested that EPO levels are one of the first humoral reactions to high altitude, with a time lag of a few hours (23). This hypothesis is substantiated by our data of significant EPO increases already after 12 h in simulated high altitude. Thirdly, after days to weeks the increase of ventilation comes to a maximum, alongside with increases in red blood cell counts and hemoglobin (23).

Analyzing the link of EPO levels to NfL leads to the probably most important finding of our study. Individuals with a higher increase in EPO concentrations showed lower pNfL levels after 12 h in simulated high altitude. Vice versa, lower EPO increase was associated with higher pNfL. This finding has to be contextualized with the discussion on the potential neuroprotective role of EPO. NfL is an established surrogate for neuroaxonal damage (16). Therefore, one might hypothesize that subjects with higher EPO levels after 12 h of simulated high-altitude exposure experience less neuroaxonal damage. A possible explanation for a neuroprotective effect of EPO is that EPO shows anti-apoptotic, anti-inflammatory, and antioxidant effects in neural tissue (28). In response to reduced oxygen availability, hypoxia-inducible factors—particularly HIF-1α—are activated as key transcriptional regulators. Among their various target genes, EPO is one of the most prominent (29). EPO binds to its receptor, expressed also on neurons, astrocytes and endothelial cells, and leads to activation of downstream signaling cascades such as the Janus kinase 2 (JAK2/STAT5) or the Mitogen-activated protein kinase (MAPK/ERK) pathways (30, 31). These pathways are involved in promoting neuronal survival, reducing oxidative stress, and stabilizing the blood–brain barrier (32). It has been demonstrated that EPO reduces glutamate toxicity, inhibits caspase-mediated apoptosis, and promotes the expression of neurotrophic factors (33, 34). Taken together, these effects could reduce neuroaxonal injury under hypoxic conditions. When neuroaxonal damage occurs in hypoxia, it is thought to be caused by mitochondrial dysfunction and increased production of reactive oxygen species. This can lead to structural damage of the neuronal cytoskeleton and consequently to degradation of neurofilaments, particularly of the light chain protein (i.e., NfL), by calcium-dependent proteases such as calpain. Subsequently, neurofilaments are released into the extracellular space and are reflected by elevated NfL levels in cerebrospinal fluid and blood (16).

It has to be pointed out, that a change in pNfL by high altitude exposure shows a peak significantly later than after 12 h, i.e., after weeks to months (19, 35–37). One might hypothesize, that the inverse correlation between EPO and NfL might be even stronger after longer follow-up. However, due to insufficient sample volume (19), we were not able to determine EPO levels at later time points. This quite short study duration restricts our ability to evaluate a potential, long-term neuroprotective effects of EPO under sustained hypoxic conditions. It is a limitation of our study leaving an interesting field for future research.

Of note, our findings show that EPO levels correlate with the extent of heart rate increase after 3 h in simulated high altitude. Increasing heart rates are a well-known phenomenon occurring nearly immediately after sudden exposure to high altitude (23). Indeed, it is known that the degree of hypoxia and resulting hypoxemia is the key driver of EPO production in the kidneys; hypoxemia in turn is correlated with the degree of heart rate increase (38, 39). Herein our findings are in line with earlier studies suggesting that EPO increases are predictable early after simulated high altitude (i.e., after 3 h in our cohort) by assessing vital parameters like the heart rate increase or the SaO2 decrease. This may have implications in both, sports medicine, for strategical high altitude training and neurological risk assessment of high altitude exposure.

In regard of increasing EPO levels the lacking increase of HIF-1α between M1 and M2 has to be pointed out. Indeed, increasing HIF-1α levels are known to usually precede EPO increases (11). The lacking increase of HIF-1α in our cohort may be explained by the timing of M2 in our cohort (i.e., after 12 h). HIF-1 is thought to mediate the acute adaption to high altitude, i.e., to hypoxia, while HIF-2 is essential in chronic hypoxia. This transition from HIF-1 to HIF-2 is called the “HIF switch” and probably occurs within hours after initiation of hypoxia (40). More precisely, it has recently been reported that HIF-1 expression is maximal after 4 h of hypoxia and is reduced dramatically by hour 8 (29). Therefore, the timing of our second measurement may have been too late, to detect the initial HIF-1α increase, as the HIF switch has already been completed.

Some limitations of this study have to be acknowledged. A first limitation may arise from the fact that not actual high-altitude exposure was tested, but simulated high altitude exposure. The simulation was done by means of a hypoxic chamber, using normobaric hypoxia. In general, normobaric hypoxia has been proposed to be a valid model of high altitude exposure (41). Nevertheless, we cannot exclude that reactions of body fluid biomarkers including EPO and NfL to actual high altitude exposure may differ from our findings. Further sources of influence may include extreme weather impact, physical exhaustion, lack of hydration or nutrition. On the other hand these confounding factors were ruled out systematically. Furthermore, we want to highlight, that our study was not designed to provide direct experimental evidence linking EPO to reductions in NfL levels, or even a causal relationship between both. To address this, future studies could involve interventional designs—both in preclinical models and clinical trials—measuring NfL levels before and after EPO treatment under controlled hypoxic or neuroinflammatory conditions. Finally, the absolute pNfL increases and differences between the two EPO groups were small. While the clinical relevance of our findings can be questioned, this was an exploratory study focusing on pathophysiological changes.

In summary, in this exploratory study we showed that EPO levels increase after simulated high altitude exposure, correlate with the increase of heart rate and the decrease of SaO2 after 3 h and that higher EPO levels are associated with lower NfL levels. Further studies including a larger number of participants and longer follow-up are needed to replicate our findings.

## Data Availability

The raw data supporting the conclusions of this article will be made available by the authors, without undue reservation.

## References

[1] Simonson TS. Altitude adaptation: a glimpse through various lenses. High Alt Med Biol. (2015) 16:125–37. doi: 10.1089/ham.2015.0033, PMID: 26070057 PMC4490743

[2] Beall CM. Andean, Tibetan, and Ethiopian patterns of adaptation to high-altitude hypoxia. Integr Comp Biol. (2006) 46:18–24. doi: 10.1093/icb/icj004, PMID: 21672719

[3] Heinicke K Prommer N Cajigal J Viola T Behn C Schmidt W. Long-term exposure to intermittent hypoxia results in increased hemoglobin mass, reduced plasma volume, and elevated erythropoietin plasma levels in man. Eur J Appl Physiol. (2003) 88:535–43. doi: 10.1007/s00421-002-0732-z, PMID: 12560952

[4] Mairer K Wille M Bucher T Burtscher M. Prevalence of acute mountain sickness in the eastern Alps. High Alt Med Biol. (2009) 10:239–45. doi: 10.1089/ham.2008.1091, PMID: 19775213

[5] Schneider M Bernasch D Weymann J Holle R Bartsch P. Acute mountain sickness: influence of susceptibility, preexposure, and ascent rate. Med Sci Sports Exerc. (2002) 34:1886–91. doi: 10.1097/00005768-200212000-00005, PMID: 12471292

[6] Luks AM Swenson ER Bärtsch P. Acute high-altitude sickness. Eur Respir Rev. (2017) 26:160096. doi: 10.1183/16000617.0096-2016, PMID: 28143879 PMC9488514

[7] Hackett PH Roach RC. High-altitude illness. N Engl J Med. (2001) 345:107–14. doi: 10.1056/NEJM200107123450206, PMID: 11450659

[8] Pugh CW Ratcliffe PJ. New horizons in hypoxia signaling pathways. Exp Cell Res. (2017) 356:116–21. doi: 10.1016/j.yexcr.2017.03.008, PMID: 28315322 PMC5653532

[9] Nairz M Schroll A Moschen AR Sonnweber T Theurl M Theurl I. Erythropoietin contrastingly affects bacterial infection and experimental colitis by inhibiting nuclear factor-κB-inducible immune pathways. Immunity. (2011) 34:61–74. doi: 10.1016/j.immuni.2011.01.002, PMID: 21256055 PMC3032045

[10] Villa P Bigini P Mennini T Agnello D Laragione T Cagnotto A. Erythropoietin selectively attenuates cytokine production and inflammation in cerebral ischemia by targeting neuronal apoptosis. J Exp Med. (2003) 198:971–5. doi: 10.1084/jem.20021067, PMID: 12975460 PMC2194205

[11] Suresh S Rajvanshi PK Noguchi CT. The many facets of erythropoietin physiologic and metabolic response. Front Physiol. (2019) 10:1534. doi: 10.3389/fphys.2019.01534, PMID: 32038269 PMC6984352

[12] Iwai M Cao G Yin W Stetler RA Liu J Chen J. Erythropoietin promotes neuronal replacement through revascularization and neurogenesis after neonatal hypoxia/ischemia in rats. Stroke. (2007) 38:2795–803. doi: 10.1161/STROKEAHA.107.483008, PMID: 17702962

[13] Bernaudin M Marti HH Roussel S Divoux D Nouvelot A MacKenzie ET. A potential role for erythropoietin in focal permanent cerebral ischemia in mice. J Cereb Blood Flow Metab. (1999) 19:643–51. doi: 10.1097/00004647-199906000-00007, PMID: 10366194

[14] Ehrenreich H Kästner A Weissenborn K Streeter J Sperling S Wang KK. Circulating damage marker profiles support a neuroprotective effect of erythropoietin in ischemic stroke patients. Mol Med. (2011) 17:1306–10. doi: 10.2119/molmed.2011.00259, PMID: 21912808 PMC3321813

[15] Ehrenreich H Weissenborn K Prange H Schneider D Weimar C Wartenberg K. Recombinant human erythropoietin in the treatment of acute ischemic stroke. Stroke. (2009) 40:e647–56. doi: 10.1161/STROKEAHA.109.564872, PMID: 19834012

[16] Khalil M Teunissen CE Otto M Piehl F Sormani MP Gattringer T. Neurofilaments as biomarkers in neurological disorders. Nat Rev Neurol. (2018) 14:577–89. doi: 10.1038/s41582-018-0058-z, PMID: 30171200

[17] Hoiland RL Ainslie PN Wellington CL Cooper J Stukas S Thiara S. Brain hypoxia is associated with neuroglial injury in humans post-cardiac arrest. Circ Res. (2021) 129:583–97. doi: 10.1161/CIRCRESAHA.121.319157, PMID: 34287000 PMC8376277

[18] Treml B Kleinsasser A Hell T Knotzer H Wille M Burtscher M. Carry-over quality of pre-acclimatization to altitude elicited by intermittent hypoxia: a participant-blinded, randomized controlled trial on antedated acclimatization to altitude. Front Physiol. (2020) 11:531. doi: 10.3389/fphys.2020.00531, PMID: 32547414 PMC7272681

[19] Berek K Lindner A Pauli FD Bsteh G Treml B Ponleitner M. Neurofilament light chain is associated with Acute Mountain sickness. Brain Behav. (2024) 14:e70165. doi: 10.1002/brb3.70165, PMID: 39552103 PMC11570677

[20] Roach RC Hackett PH Oelz O Bärtsch P Luks AM MacInnis MJ. The 2018 Lake Louise acute mountain sickness score. High Alt Med Biol. (2018) 19:4–6. doi: 10.1089/ham.2017.0164, PMID: 29583031 PMC6191821

[21] Rissin DM Kan CW Campbell TG Howes SC Fournier DR Song L. Single-molecule enzyme-linked immunosorbent assay detects serum proteins at subfemtomolar concentrations. Nat Biotechnol. (2010) 28:595–9. doi: 10.1038/nbt.1641, PMID: 20495550 PMC2919230

[22] Benkert P Meier S Schaedelin S Manouchehrinia A Yaldizli Ö Maceski A. Serum neurofilament light chain for individual prognostication of disease activity in people with multiple sclerosis: a retrospective modelling and validation study. Lancet Neurol. (2022) 21:246–57. doi: 10.1016/S1474-4422(22)00009-6, PMID: 35182510

[23] Luks AM Hackett PH. Medical conditions and high-altitude travel. N Engl J Med. (2022) 386:364–73. doi: 10.1056/NEJMra2104829, PMID: 35081281

[24] Eltzschig HK Carmeliet P. Hypoxia and inflammation. N Engl J Med. (2011) 364:656–65. doi: 10.1056/NEJMra0910283, PMID: 21323543 PMC3930928

[25] Hagemeyer N Boretius S Ott C Von Streitberg A Welpinghus H Sperling S. Erythropoietin attenuates neurological and histological consequences of toxic demyelination in mice. Mol Med. (2012) 18:628–35. doi: 10.2119/molmed.2011.00457, PMID: 22396019 PMC3388128

[26] Ostrowski D Ehrenreich H Heinrich R. Erythropoietin promotes survival and regeneration of insect neurons in vivo and in vitro. Neuroscience. (2011) 188:95–108. doi: 10.1016/j.neuroscience.2011.05.018, PMID: 21600963

[27] Sättler MB Merkler D Maier K Stadelmann C Ehrenreich H Bähr M. Neuroprotective effects and intracellular signaling pathways of erythropoietin in a rat model of multiple sclerosis. Cell Death Differ. (2004) 11:S181–92. doi: 10.1038/sj.cdd.4401504, PMID: 15459752

[28] Byts N Sirén AL. Erythropoietin: a multimodal neuroprotective agent. Exp Transl Stroke Med. (2009) 1:4. doi: 10.1186/2040-7378-1-4, PMID: 20142991 PMC2816866

[29] Jaśkiewicz M Moszyńska A Króliczewski J Cabaj A Bartoszewska S Charzyńska A. The transition from HIF-1 to HIF-2 during prolonged hypoxia results from reactivation of PHDs and HIF1A mRNA instability. Cell Mol Biol Lett. (2022) 27:109. doi: 10.1186/s11658-022-00408-7, PMID: 36482296 PMC9730601

[30] Brines ML Ghezzi P Keenan S Agnello D de Lanerolle NC Cerami C. Erythropoietin crosses the blood-brain barrier to protect against experimental brain injury. Proc Natl Acad Sci USA. (2000) 97:10526–31. doi: 10.1073/pnas.97.19.10526, PMID: 10984541 PMC27058

[31] Digicaylioglu M Lipton SA. Erythropoietin-mediated neuroprotection involves cross-talk between Jak2 and NF-kappaB signalling cascades. Nature. (2001) 412:641–7. doi: 10.1038/35088074, PMID: 11493922

[32] Mallet RT Ryou MG. Erythropoietin: endogenous protection of ischemic brain. Vitam Horm. (2017) 105:197–232. doi: 10.1016/bs.vh.2017.01.002, PMID: 28629519

[33] Morishita E Masuda S Nagao M Yasuda Y Sasaki R. Erythropoietin receptor is expressed in rat hippocampal and cerebral cortical neurons, and erythropoietin prevents in vitro glutamate-induced neuronal death. Neuroscience. (1997) 76:105–16. doi: 10.1016/S0306-4522(96)00306-5, PMID: 8971763

[34] Wang L Zhang Z Wang Y Zhang R Chopp M. Treatment of stroke with erythropoietin enhances neurogenesis and angiogenesis and improves neurological function in rats. Stroke. (2004) 35:1732–7. doi: 10.1161/01.STR.0000132196.49028.a4, PMID: 15178821

[35] Pedersen A Stanne TM Nilsson S Klasson S Rosengren L Holmegaard L. Circulating neurofilament light in ischemic stroke: temporal profile and outcome prediction. J Neurol. (2019) 266:2796–806. doi: 10.1007/s00415-019-09477-9, PMID: 31375988 PMC6803587

[36] Tiedt S Duering M Barro C Kaya AG Boeck J Bode FJ. Serum neurofilament light: a biomarker of neuroaxonal injury after ischemic stroke. Neurology. (2018) 91:e1338–47. doi: 10.1212/WNL.0000000000006282, PMID: 30217937

[37] Gattringer T Pinter D Enzinger C Seifert-Held T Kneihsl M Fandler S. Serum neurofilament light is sensitive to active cerebral small vessel disease. Neurology. (2017) 89:2108–14. doi: 10.1212/WNL.0000000000004645, PMID: 29046363 PMC5711505

[38] Jelkmann W. Regulation of erythropoietin production. J Physiol. (2011) 589:1251–8. doi: 10.1113/jphysiol.2010.195057, PMID: 21078592 PMC3082088

[39] Saito S Tanobe K Yamada M Nishihara F. Relationship between arterial oxygen saturation and heart rate variability at high altitudes. Am J Emerg Med. (2005) 23:8–12. doi: 10.1016/j.ajem.2004.09.023, PMID: 15672330

[40] Serocki M Bartoszewska S Janaszak-Jasiecka A Ochocka RJ Collawn JF Bartoszewski R. miRNAs regulate the HIF switch during hypoxia: a novel therapeutic target. Angiogenesis. (2018) 21:183–202. doi: 10.1007/s10456-018-9600-2, PMID: 29383635 PMC5878208

[41] Burtscher M Flatz M Faulhaber M. Prediction of susceptibility to acute mountain sickness by SaO2 values during short-term exposure to hypoxia. High Alt Med Biol. (2004) 5:335–40. doi: 10.1089/ham.2004.5.335, PMID: 15453999

